# An In Vivo (*Gallus gallus*) Feeding Trial Demonstrating the Enhanced Iron Bioavailability Properties of the Fast Cooking Manteca Yellow Bean (*Phaseolus vulgaris* L.)

**DOI:** 10.3390/nu11081768

**Published:** 2019-08-01

**Authors:** Jason A. Wiesinger, Raymond P. Glahn, Karen A. Cichy, Nikolai Kolba, Jonathan J. Hart, Elad Tako

**Affiliations:** 1 USDA-ARS, Robert W. Holley Center for Agriculture and Health, Cornell University, Ithaca, NY 14853, USA; 2 USDA-ARS, Sugarbeet and Bean Research, Michigan State University, East Lansing, MI 48824, USA

**Keywords:** *Phaseolus vulgaris* L., yellow bean, cooking time, iron, iron bioavailability, phytate, polyphenols, kaempferol 3-glucoside, Caco-2 cell bioassay, *Gallus gallus*

## Abstract

The common dry bean (*Phaseolus vulgaris* L.) is a globally produced pulse crop and an important source of micronutrients for millions of people across Latin America and Africa. Many of the preferred black and red seed types in these regions have seed coat polyphenols that inhibit the absorption of iron. Yellow beans are distinct from other market classes because they accumulate the antioxidant kaempferol 3-glucoside in their seed coats. Due to their fast cooking tendencies, yellow beans are often marketed at premium prices in the same geographical regions where dietary iron deficiency is a major health concern. Hence, this study compared the iron bioavailability of three faster cooking yellow beans with contrasting seed coat colors from Africa (Manteca, Amarillo, and Njano) to slower cooking white and red kidney commercial varieties. Iron status and iron bioavailability was assessed by the capacity of a bean based diet to generate and maintain total body hemoglobin iron (Hb-Fe) during a 6 week in vivo (*Gallus gallus*) feeding trial. Over the course of the experiment, animals fed yellow bean diets had significantly (*p* ≤ 0.05) higher Hb-Fe than animals fed the white or red kidney bean diet. This study shows that the Manteca yellow bean possess a rare combination of biochemical traits that result in faster cooking times and improved iron bioavailability. The Manteca yellow bean is worthy of germplasm enhancement to address iron deficiency in regions where beans are consumed as a dietary staple.

## 1. Introduction

The common dry bean (*Phaseolus vulgaris* L.) is a globally produced pulse crop that serves as an important source of protein and micronutrients for millions of people across Africa, the Caribbean, Latin America, and Southern Europe [1]. Dry beans accumulate trace minerals, such as iron and zinc, into their seed by using a complex network of ion transporters and chelating molecules that include phytate, nicotianamine, and polyphenols [2]. Traditional breeding practices can be used to generate bean seeds with very high iron concentrations [3]. This lead to the conception of using biofortified bean varieties as a vehicle to alleviate trace mineral deficiencies in resource-limited regions of Latin America and Sub-Saharan Africa, where beans are widely accepted as a dietary staple [4]. Many of the preferred seed types in these regions are black and red beans, which contain polyphenolic compounds that inhibit the absorption of iron in the upper intestine, potentially limiting their nutritional impact [5,6,7,8]. Polyphenols and other prebiotic molecules that survive enzymatic digestion in the small intestine, however, can stimulate the growth of beneficial microbiota in the lower intestine, improving the overall health of the digestive system [9,10,11,12,13,14].

Iron uptake assays in Caco-2 cells indicate that not all polyphenols are inhibitors of iron absorption [7]. Certain polyphenolic compounds, such as kaempferol and kaempferol 3-glucoside are shown to promote iron uptake in vitro [5,7]. Kaempferol is a flavonoid expressed in the seed coats of many bean seed types including black, carioca, cranberry, pinto, kidney, and small red [15,16]. While the concentrations of kaempferol compounds vary between the different bean market classes, the most dominant polyphenol measured in seed coats of yellow beans is kaempferol 3-glucoside [17,18]. Alleles in the color genes of yellow beans direct polyphenol pathways in the seed coat to create a vast array of color combinations that range from bright neon yellow to orange and green [19,20]. 

Originating from the Peruano coast, yellow beans were cultivated very early in human history [21] and over the centuries have diversified into many different market classes that are sold throughout Central and South America, as well as Sub-Saharan Africa [22,23]. Yellow beans owe their long heritage to smallholder farmers selecting for unique seed coat colors that would appeal to consumers at the marketplace, and are often marketed at premium prices in Africa for their fast cooking tendencies [23,24]. Iron uptake studies measuring the formation of ferritin protein in Caco-2 cells recently showed that a set of fast cooking yellow beans from Africa have more bioavailable iron than slower cooking yellow and red mottled beans from Africa and the Caribbean [25]. Exploring the yellow bean’s unique market classes to develop new fast cooking varieties that could potentially deliver more absorbable iron would be a useful strategy; especially for regions where long cooking times often deter consumers purchasing beans and where micronutrient deficiencies, such as iron deficiency anemia are highly prevalent [26,27].

The purpose of this research was to further evaluate the iron nutrition and iron bioavailability of different yellow bean market classes by incorporating cooked beans into diets for a long-term in vivo feeding trial. The objective of this study was to compare the nutritional properties, polyphenolic profiles, and iron bioavailability of three yellow beans with contrasting seed coat colors from Africa (Manteca, Amarillo, Njano) to slower cooking white and red kidney commercial varieties from North America. Bean based diets were formulated with cooked beans as the major ingredient and included the complementary food crops of potato, rice, and cabbage. Iron bioavailability was evaluated for each of bean based diets with a Caco-2 bioassay and by the ability to maintain total body hemoglobin iron (Hb-Fe) during a 6 week in vivo (*Gallus gallus*) feeding trial.

## 2. Materials and Methods 

### 2.1. Plant Materials—African Yellow Beans and Commercial Kidney Bean Varieties

**Figure 1:**
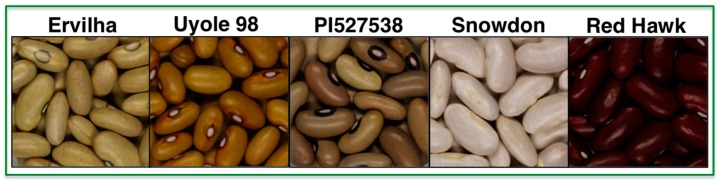
Photographs depicting the five genotypes used to evaluate the iron bioavailability of the African yellow bean. To compare differences in seed sizes, all photographs were taking to scale under standardized lighting conditions.

Three yellow and two kidney *P. vulgaris* genotypes with contrasting seed coat colors were selected for this study. Ervilha is a fast cooking Manteca (pale-lemon) landrace collected from the Instituto de Investigação Agronómica located in the Huambo province of Angola [25]. Uyole 98 is an Amarillo (yellow-orange) variety release by the Tanzanian Breeding Program in 1999, renowned for its strong agronomic performance, disease resistance, and consumer quality traits, such as fast cooking times and excellent taste [28]. PI527538 is a Njano (yellow-green) landrace collected from Burundi. Selected for generations among farmers throughout Sub-Saharan Africa, the Njano and Soya Njano market classes are preferred seed types among consumers in East Africa [29]. Snowdon is a white kidney bean released by Michigan State University AgBioResearch in 2012. Snowdon is an early maturing, disease tolerant white bean with good canning qualities [30]. Red Hawk is a dark red kidney variety jointly released by Michigan State University and USDA-ARS in 1998. Red Hawk is an early maturing, disease-resistant red kidney bean with excellent canning and processing qualities [31]. Photographs depicting the different seed coat colors of each bean are shown in Figure 1. A summary of the collection sites, sources and cultivation status of each genotype is presented in Table 1.

### 2.2. Field Design, Growing Conditions and Post Harvest Handling

Genotypes were planted side-by-side with 0.5 m spacing between rows at the Michigan State University Montcalm Research Farm near Entrican, MI in 2017. The soil type at the Montcalm Research Farm is Eutric Glossoboralfs (coarse-loamy, mixed) and Alfic Fragiorthods (coarse-loamy, mixed, frigid). Rainfall was supplemented with overhead irrigation as needed. Weeds and pests were controlled by hand or with herbicides if needed. Upon maturity, bean plants were pulled by hand and then threshed with a Hege 140 plot harvester (Wintersteiger Inc., Salt Lake City, UT, USA). Immediately after harvest, seeds were hand sorted to eliminate any immature, wrinkled, discolored, or damaged seeds. To equilibrate moisture content, sorted seed was placed into dark storage under ambient conditions (20–22 °C, 50–60% relative humidity) at standard atmospheric pressure and monitored for eight weeks with a John Deere Grain Moisture Tester (Moisture Chek-Plus™; Deere & Company, Watseka, IL, USA) until the moisture content reached 10–12% [32].

### 2.3. Cooking Time Determination

Growing conditions and post-harvest handling ensured the differences in cooking times between the yellow and kidney beans were under genetic control and not influenced by the growing or processing conditions of the seed after harvest [25]. Subsets of 50 seed from four randomly selected field replicates for each genotype were evaluated for cooking time. Prior to cooking, bean seeds were pre-soaked in distilled water (1:6 *w*/*w*) for 12 h at room temperature. Cooking time was measured with a Mattson pin drop cooking device, which fits into a 4 L stainless steel beaker heated over an electric portable burner containing 1.8 L of boiling distilled water. Cooking time was standardized as the number of minutes required for twenty out of twenty-five piercing tip rods (70 g, 2 mm diameter) to pass completely through each seed under a steady boil at 100 °C [33].

### 2.4. Ingredient Preparation and Diet Composition

To prepare beans for diet formulation, 35 kg of raw seed were first rinsed and cleaned thoroughly in distilled water to remove dust, debris, and non-edible material. Beans were pre-soaked in distilled water (1:6 *w*/*w*) for 12 h at room temperature before cooking at the Food Processing and Development Laboratory (FPDL) located at Cornell University, Ithaca, New York. Beans were cooked according to their predetermined cooking times in boiling distilled water using large (20 gallon) stainless steel steam kettles at the processing facility. Drained beans were spread evenly in stainless steel trays and allowed to cool for thirty minutes at room temperature. Cooked beans were then stored in a −20 °C cold room for 24 h prior to freeze-drying (VirTis Research Equipment, Gardiner, NY, USA). Basmati rice was purchased from Wegmans™ food store located in Ithaca, NY, USA. Large quantities of rice (25 kg) were cooked with distilled water at the FPDL in stainless steel steam kettles. Cooked rice was cooled to room temperature on stainless steel trays and stored at −20 °C for 24 h before freeze-drying. Cooked/air-dried potatoes and white cabbage were purchased from North Bay Trading Co. (Brule, WI, USA). Dried ingredients were milled into a course powder using a Waring Commercial^®^ CB15 stainless steel blender (Torrington, CT, USA). Chick Vitamin Mixture 330,002 and Salt Mix for Chick Diet 230,000 (without iron) was purchased from Dyets Inc. (Bethlehem, PA, USA). DL-Methionine and choline chloride were purchased from Sigma–Aldrich (St. Louis, MO, USA). Ingredients were mixed with corn oil (0.5 L/kg) and stored at 4 °C during the duration of the feeding trial. The final composition of each bean based diet is presented in Table 2.

### 2.5. Iron Analysis

For iron analysis, either a 500 mg sample of each ingredient, a 500 mg sample of each bean based diet or a 100 mg sample of liver tissue (wet weight) were pre-digested in boro–silicate glass tubes with 3 mL of a concentrated ultra-pure nitric acid and perchloric acid mixture (60:40 *v*/*v*) for 16 h at room temperature. Samples were then placed in a digestion block (Martin Machine, Ivesdale, IL, USA) and heated incrementally over 4 h to a temperature of 120 °C with refluxing. After incubating at 120 °C for 2 h, 2 mL of concentrated ultra-pure nitric acid was subsequently added to each sample before raising the digestion block temperature to 145 °C for an additional 2 h. The temperature of the digestion block was then raised to 190 °C and maintained for at least ten minutes before samples were allowed to cool at room temperature. Digested samples were re-suspended in 20 mL of ultrapure water prior to analysis using ICP-AES (inductively coupled plasma-atomic emission spectroscopy; Thermo iCAP 6500 Series, Thermo Scientific, Cambridge, UK) with quality control standards (High Purity Standards, Charleston, SC, USA) following every 10 samples. Yttrium purchased from High Purity Standards (10M67-1) was used as an internal standard. All samples were digested and measured with 0.5 μg/mL of Yttrium (final concentration) to ensure batch-to-batch accuracy and to correct for matrix inference during digestion.

### 2.6. Phytate Analysis

For phytate (phytic acid) determination, a 500 mg sample from each ingredient and a 500 mg sample from each of the bean based diets were first extracted in 10 mL of 0.66 M hydrochloric acid under constant motion for 16 h at room temperature. A 1 mL aliquot of total extract was collected using a wide bore pipet tip, and then centrifuged (16,000 *g*) for 10 min to pellet debris. A 0.5 mL sample of supernatant was then neutralized with 0.5 mL 0.75 M sodium hydroxide and stored at −20 °C until the day of analysis. A phytate/total phosphorous kit (K-PHYT; Megazyme International, Bray, Ireland) was used to measure liberated phosphorous by phytase and alkaline phosphatase. Phosphorous was quantified by colorimetric analysis as molybdenum blue with phosphorous standards read at a wavelength of 655 nm against the absorbance of a reagent blank. Total phytate concentrations were calculated with Mega-Calc™ by subtracting free phosphate concentrations in the extracts from the total amount of phosphorous that is exclusively released after enzymatic digestion.

### 2.7. Protein and Fiber Analysis

Total nitrogen concentrations were measured in a 500 mg sample of cooked beans by the Dumas combustion method at A&L Great Lakes Laboratories (Fort Wayne, IN, USA) in accordance with AOAC method 968.06 [34]. The percentage of crude protein was estimated by multiplying the dry weight total nitrogen concentration by a factor of 6.25 [35]. Insoluble, soluble and total fiber concentrations were determined by the enzymatic-gravimetric AOAC method 985.29 [36], using enzymatic hydrolysis with heat-resistant amylase, protease, and amyloglucosidase (Total Dietary Fiber Assay Kit, Sigma Aldrich Co., St. Louis, MO, USA).

### 2.8. Polyphenolic Extraction

For polyphenol extraction, 5 mL of methanol:water (50:50 *v*/*v*) was added to either 500 mg of cooked beans or to 500 mg of bean based diet, and vortexed for one minute before incubating in a sonication water bath for 20 min at room temperature. Samples were again vortexed and placed on a compact digital Rocker (Labnet International, Inc., Edison, NJ, USA) at room temperature for 60 min before centrifuging at 4000 *g* for 15 min. Supernatants were filtered with a 0.2 μm Teflon™ syringe filter and stored at −20 °C until chemical analysis.

#### Liquid Chromatography—Mass Spectrometry (LC–MS) Analysis of Polyphenols

Extracts and standards were analyzed by an Agilent 1220 Infinity Liquid Chromatograph (LC; Agilent Technologies, Inc., Santa Clara, CA, USA) coupled to an Advion expressionL^®^ compact mass spectrometer (CMS; Advion Inc., Ithaca, NY, USA). Two-μL samples were injected and passed through an Acquity™ UPLC BEH Shield RP18 1.7 µm 2.1 × 100 mm column (Waters, Milford, MA, USA) at 0.35 mL/min. The column was temperature-controlled at 45 °C. The mobile phase consisted of ultra-pure water with 0.10% formic acid (solvent A) and acetonitrile with 0.10% formic acid (solvent B). Polyphenols were eluted using linear gradients of 86.7 to 77.0% A in 0.50 min, 77.0 to 46.0% A in 5.50 min, 46.0 to 0% A in 0.50 min, held at 0% A for 3.50 min, 0 to 86.7% A in 0.50 min, and held at 86.7% A for 3.50 min, for a total run time of 14 min. From the column, flow was directed into a variable wavelength UV detector set at 278 nm. Flow was then directed into the source of an Advion expressionL^®^ CMS, and electro spray ionization (ESI) mass spectrometry was performed in negative ionization mode using selected ion monitoring with a scan time of 50 milliseconds for the 18 polyphenol masses of interest. Capillary temperature and voltages were 300 °C and 100 volts, respectively. ESI source voltage and gas temperature were 2.6 kilovolts and 240 °C respectively. Desolvation gas flow was 240 L/h. Advion Mass Express™ software was used to control the LC and CMS instrumentation and data acquisition. Individual polyphenols were identified and confirmed by comparison of *m*/*z* and LC retention times with authentic standards. Polyphenol standard curves for flavonoids were derived from integrated areas under UV absorption peaks from 8 replications. Standard curves for catechin and 3.4-dihydroxybenzoic acid were constructed from MS ion intensities using 8 replications.

### 2.9. In Vitro Iron Bioavailability Assessment (Caco-2 Cell Bioassay)

An established in vitro digestion/Caco-2 cell culture model was initially used to assess the iron bioavailability of cooked beans and bean based diets [37,38,39]. A 500 mg sample of lyophilized powder from either whole cooked beans or bean based diets were subjected to a simulated gastric and intestinal digestion as described previously [37,39]. The bioassay was performed according to the detailed methods described in Glahn et al., 1998 [37]. The bioassay works according to the following principle. In response to increases in cellular iron concentrations, Caco-2 cells produce more ferritin protein. Therefore, iron bioavailability was determined as the increase in Caco-2 cell ferritin production expressed as a ratio to total protein (ng ferritin per mg of total cell protein) after exposure to a digested sample [37,38,39]. Ferritin was measured by enzyme linked immunoassay (Human Ferritin ELISA kit S-22, Ramco Laboratories Inc., Stafford, TX, USA) and total cell protein concentrations were quantified using the Bio-Rad DC™ protein assay kit (Bio-Rad Laboratories Inc., Hercules, CA, USA). 

To confirm the responsiveness of the bioassay, each experiment was run with several quality controls. These include a blank-digest, which is only the physiologically balanced saline and the gastrointestinal enzymes. The blank-digest was used to ensure there was no iron contamination in the bioassay. Ferritin values of Caco-2 cells were exposed to the blank-digest averaged 2.5 ± 0.08 ng ferritin/mg cell protein (mean ± Standard Deviation; SD) over the course of two cell culture experiments. The responsiveness of the bioassay was monitored by: (1) a blank-digest with FeCl_3_ (66 μM) and (2) a blank-digest of FeCl_3_ (66 μM) plus the addition of 1.3 mM ascorbic acid (Sigma Aldrich Co., St. Louis, MO, USA). Ferritin values for the FeCl_3_ digest and the FeCl_3_ digest with ascorbic acid averaged 68 ± 5.3 and 234 ± 17 ng/mg cell protein (mean ± SD), respectively. The quality controls could be utilized as a reference standard because they do not contain the same food matrix properties as the cooked beans. Therefore, a cooked/lyophilized/milled navy bean control (commercial variety Merlin) was run with each assay as a reference standard to index the ferritin/total cell protein ratios of the Caco-2 cells over the course of several cell culture experiments. Ferritin values for the Merlin navy bean control averaged 8.07 ± 0.63 ng/mg cell protein (mean ± SD).

### 2.10. Animals and Feeding Trial Design

Cornish-cross fertile broiler eggs were obtained from a commercial hatchery (Moyer’s Chicks, Quakertown, PA, USA). The eggs were incubated under optimal conditions at the Cornell University Animal Science poultry farm incubator. On the day of hatching (hatchability = 95%) chicks were randomly allocated into five treatment groups (*n* = 13) shown in Table 2 and given ad libitum access to food and water (iron concentration <0.4 µg/L). Chicks were housed in a total confinement building (3–4 animals per 1 m^2^ metal cage) under controlled temperatures and humidity with 16 h of light. Each cage was equipped with an automatic watering dish and a manual self-feeder. Feed intakes were measured daily starting from the day of hatching and body weights were recorded each week. All animal protocols were approved by the Cornell University Institutional Animal Care and Use Committee (protocol# 2007-0129).

### 2.11. Blood Collection, Hemoglobin and Index of Iron Absorption Calculations

Blood samples were collected from the wing vein of each animal with heparinized capillary tubes and stored on ice in BD Vacutainer^®^ vials (lithium heparin; 95 USP Units). For Hemoglobin (Hb) determination, whole blood samples were diluted 100-fold in distilled water and measured colorimetrically with BioAssay Systems Quantichrom™ hemoglobin assay (DIHB-250) following the manufacturer’s instructions (BioAssay Systems, Hayward, CA, USA). Total body hemoglobin iron (Hb-Fe) is an index of iron absorption, and is calculated from hemoglobin concentrations and total blood volume based on body weight (85 mL per kg of body weight) [6,40,41,42]:

Hb-Fe (mg) = Body Weight (kg) × 0.085 L/kg × Hb (g/L) × 3.35 mg Fe/g Hb.(1)

Hemoglobin maintenance efficiency (HME) is calculated as the cumulative difference in total body hemoglobin iron from the start of the experiment, divided by total dietary iron intake. Dietary iron intakes were determined by multiplying the cumulative amount of diet consumed over the course of the experiment with the iron concentrations measured for each diet shown in Table 2 [6,40,41,42]:

(2) $$\mathrm{HME}=\frac{\mathrm{Hb}\;\mathrm{Fe}\;(\mathrm{final})\;\mathrm{mg}-\mathrm{Hb}\;\mathrm{Fe}\;(\mathrm{initial})\;\mathrm{mg}}{\mathrm{Total}\;\mathrm{Fe}\;\mathrm{Intake}\;(\mathrm{mg})}\times [100\%].$$

At the end of the experiment (42 days), animals were euthanized by CO_2_ exposure and their blood, small intestine, cecum, and liver were quickly collected. Tissue was immediately frozen in liquid nitrogen and stored at −80 °C until further analysis.

### 2.12. Liver Iron and Ferritin

The quantification of liver Fe (ICP-AES) and liver ferritin were conducted as previously described [40,43,44]. Polyacrylamide gels were scanned with a Bio-Rad^®^ densitometer and the measurement of band intensity conducted with Quantity-One 1-D analysis program (Bio-Rad Laboratories Inc., Hercules, CA, USA). Local background was subtracted from each sample and horse spleen ferritin (Sigma Aldrich Co., St. Louis, MO, USA) was used as a standard for identifying and calibrating ferritin protein.

### 2.13. Isolation of Total RNA from Duodenum

Total RNA was extracted using Qiagen RNeasy Mini Kit (RNeasy Mini Kit, Qiagen Inc., Valencia, CA, USA) according to the manufacturer’s protocol. Proximal duodenal tissue (30 mg) was disrupted and homogenized with a rotor-stator homogenizer in buffer RLT^®^, containing β-mercaptoethanol. The tissue lysate was centrifuged for 3 min at 8000 *g* in a microcentrifuge. An aliquot of the supernatant was transferred to another tube, combined with 1 volume of 70% ethanol and mixed immediately. Each sample (700 μL) was applied to an RNeasy mini column, centrifuged for 15 s at 8000 *g*, and the flow through material was discarded. The RNeasy columns were transferred to new 2 mL collection tubes, and 500 μL of buffer RPE^®^ was pipetted onto the RNeasy column followed by centrifugation for 15 s at 8000 *g*. An additional 500 μL of buffer RPE were pipetted onto the RNeasy column and centrifuged for 2 min at 8000 *g*. Total RNA was eluted in 50 μL of RNase free water. All steps were carried out under RNase free conditions. RNA was quantified by absorbance at A 260/280. Integrity of the 28S and 18S ribosomal RNAs was verified by 1.5% agarose gel electrophoresis followed by ethidium bromide staining. DNA contamination was removed using TURBO DNase treatment and removal kit from AMBION (Austin, TX, USA).

### 2.14. Real Time Polymerase Chain Reaction (RT-PCR)

To create the cDNA, a 20 µL reverse transcriptase (RT) reaction was completed in a BioRad C1000 touch thermocycler using the Improm-II Reverse Transcriptase Kit (Catalog #A1250; Promega Corp., Madison, WI, USA). The first step consisted of 1 µg of total RNA template, 10 µM of random hexamer primers, and 2 mM of oligo-dT primers. The RT protocol was used to anneal primers to RNA at 94 °C for 5 min. The first strand was copied for 60 min at 42 °C (optimum temperature for the enzyme), followed by exposure to 70 °C (15 min) for enzymatic inactivation, samples were then held at 4 °C until quantification by a Nanodrop™ ND-1000 (Thermo Fisher Scientific, Wilmington, DE, USA). The concentration of cDNA obtained was determined by measuring the absorbance at 260 nm and 280 nm using an extinction coefficient of 33 (for single stranded DNA). Genomic DNA contamination was assessed by a real-time RT-PCR assay for the reference gene samples.

### 2.15. Primer Design for Divalent Metal Transporter 1, Duodenal Cytochrome B, and Ferroportin Gene Expression Analysis

The primers used in the real-time PCR to measure the duodenum gene expression of the iron import proteins divalent metal transporter 1 (DMT-1) and duodenal cytochrome B (DcytB), as well as the iron export protein ferroportin were designed according gene sequences obtained from the National Center for Biotechnology Information (NCBI) Genbank^®^ database, using Real-Time Primer Design Tool software (IDT DNA, Coralvilla, IA, USA). The sequences and the description of the forward and reverse primers used for PCR reactions in this study are summarized in Table S1. The amplicon length was limited to 90 to 150 base pairs. The length of the primers was 17–25 bp, and the GC content was between 41% and 55%. The *Gallus gallus* primer 18S Ribosomal subunit 18S rRNA was designed as a reference gene (Table S1).

### 2.16. Real-Time qPCR Design

Isolated cDNA was used for each 10 µL reaction together with 2× BioRad^®^ SSO Advanced Universal SYBR Green Supermix (Cat. #1725274, Hercules, CA, USA) which included buffer, Taq DNA polymerase, dNTPs and SYBR green dye. Specific primers (Table S1) and cDNA or water (for no template control) were added to each PCR reaction. For each gene, the optimal MgCl_2_ concentration produced the amplification plot with the lowest cycle product (Cq), the highest fluorescence intensity and the steepest amplification slope. Master mix (8 µL) was pipetted into the 96-well plate and 2 µL cDNA was added as PCR template. Each run contained seven standard curve points in duplicate. A no template control of nuclease-free water was included to exclude DNA contamination in the PCR mix. The double stranded DNA was amplified in the Bio-Rad^®^ CFX96 Touch (Hercules, CA, USA) using the following PCR conditions: initial denaturing at 95 °C for 30 s, 40 cycles of denaturing at 95 °C for 15 s, various annealing temperatures according to Integrated DNA Technologies (IDT) for 30 s and elongating at 60 °C for 30 s. The data on the expression levels of the genes were obtained as Cq values based on the “second derivative maximum” (=automated method) as computed by the software. For each of the four genes, the reactions were run in duplicate. All assays were quantified by including a standard curve in the real-time qPCR analysis. The next four points of the standard curve were prepared by a 1:10 dilution. Each point of the standard curve was included in duplicate. A graph of Cq vs. log (10) concentrations was produced by the software and the efficiencies were calculated as 10 [1/slope]. The specificity of the amplified real-time RT-PCR products was verified by melting curve analysis (60–95 °C) after 40 cycles, resulting in a number of different specific products, each with a specific melting temperature. Real-time RT-PCR efficiency (E) values for the four genes were as follows: DMT-1, 0.988; DcytB, 1.046; Ferroportin, 1.109; 18S rRNA, 0.934.

### 2.17. Collection of Microbial Samples and DNA Isolation of Intestinal Contents

The cecum was aseptically (500 mg) removed and placed into a sterile 50 mL tube containing 9 mL of sterile PBS and homogenized by vortexing with glass beads (3 mm diameter) for 3 min. Debris was removed by centrifugation at 700 *g* for 1 min, and the supernatant was collected and centrifuged at 12,000 *g* for 5 min. The pellet was washed twice with 1 × Phosphate Buffered Saline (BP399-1; Fisher Scientific, Inc., Hampton, NH, USA) and stored at −20 °C until DNA extraction. For DNA purification, the pellet was re-suspended in 50 mM Ethylenediaminetetraacetic acid (EDTA) and treated with lysozyme (Sigma Aldrich Co., St. Louis, MO, USA; final concentration of 10 mg/mL for 45 min at 37 °C. The bacterial genomic DNA was isolated using a Wizard^®^ Genomic DNA purification kit (Promega Corp., Madison, WI, USA).

### 2.18. Primers Design and PCR Amplification of Bacterial 16S rRNA

Primers for Bifidobacterium, Lactobacillus, E. coli and Clostridium were designed according to previously published data [45]. To evaluate the relative proportion of each bacterium, all targeted primers were normalized to the reference gene of the universal primer product 16S rRNA. PCR products were separated by electrophoresis on 2% agarose gel, stained with ethidium bromide, and quantified using the Quantity One 1-D analysis software (Bio-Rad, Hercules, CA, USA).

### 2.19. Statistical Analysis

All statistical analyses were conducted using IBM SPSS Statistics 25 (IBM Analytics, Armonk, NY, USA). The normality of residuals for each parameter was evaluated using the Kolmogorov-Smirnov test. Equality of variance for each parameter was determined using the Bartlett’s test. Measured parameters were found to have a normal distribution and equal variance, and were, therefore, acceptable for ANOVA without additional data transformation steps. Mean separations for measured parameters were determined using ANOVA with the model including dietary treatment (5 levels) as fixed effects; followed by a Duncan *post hoc* test. Differences with *p* values of ≤ 0.05 were considered statistically significant. Graphs were prepared using GraphPad Prism7 (GraphPad Software, La Jolla, CA, USA).

## 3. Results

### 3.1. Cooking Times and Seed Iron-Phytate Concentrations

Table 1 shows the cooking times of the yellow and kidney beans prior to diet formulation. Significant (*p* ≤ 0.05) differences in cooking times were measured among the pre-soaked beans, ranging from 15 min for Ervilha to 37 min for Red Hawk (Table 1). Significantly (*p* ≤ 0.05) faster cooking times were measured in the yellow beans when compared to the white and dark red kidney bean varieties (Table 1). Iron concentrations of each ingredient after cooking, lyophilizing, and milling are show in Table 2. Differences in seed iron concentrations between the cooked beans were significant (*p* ≤ 0.05), ranging from 75 μg/g in Snowdon to 85 μg/g in PI527538 (Table 2). Phytate concentrations and phytate molar ratios of each ingredient used to formulate the bean based diets are shown in Table S2. Significant (*p* ≤ 0.05) differences in phytate concentrations were measured between the cooked yellow and kidney beans, ranging from 12.8 mg/g in Red Hawk to 13.7 mg/g in Ervilha (Table S2). Phytate to iron molar ratios also varied significantly (*p* ≤ 0.05), ranging from a ratio of 13.4 in Red Hawk and PI527538 to a ratio of 15.3 in Snowdon (Table S2).

### 3.2. Protein and Fiber Concentrations of Cooked Beans

Table 3 shows the total crude protein concentrations of yellow and kidney beans after cooking. Significant differences (*p* ≤ 0.05) in protein concentrations were measured between the cooked beans, ranging from 21 g/100 g in Snowdon to 26 g/100 g in PI527538 (Table 3). The concentrations of insoluble, soluble, and total fiber for each of the cooked beans are also shown in Table 3. Significant differences (*p* ≤ 0.05) in each of the fiber fractions were measured among the yellow and kidney beans. The lowest concentrations of the insoluble and total fiber were detected in the fastest cooking yellow bean Ervilha (Table 3). Significantly (*p* ≤ 0.05) higher concentrations of all three fiber fractions were in measured in each of the kidney beans, when compared to the yellow beans Ervilha and PI527538 after cooking (Table 3).

### 3.3. Iron-Phytate Analysis of Bean Based Diets

The final composition including the iron concentrations, phytate concentrations, and phytate-iron molar ratios for each of the five bean based diets are shown in Table 2. Iron concentrations between the bean based diets were significantly different (*p* ≤ 0.05). Diets formulated from Ervilha, PI527538 and Red Hawk had higher iron concentrations (52–55 μg/g) than diets formulated from Uyole 98 and Snowdon (47 μg/g). Final phytate concentrations also varied between the five diets ranging from 6.91 mg/g in PI527538 to 7.55 mg/g in Ervilha (Table 2). Significant (*p* ≤ 0.05) differences in phytate-iron molar ratios were calculated between the bean based diets, ranging from 11.3 mg/g in PI527538 to 13.9 mg/g in Uyole 98 (Table 2).

### 3.4. Polyphenolic Profile of Beans and Bean Based Diets

The polyphenol concentrations of the yellow and kidney beans after cooking are shown in Table 4. Eleven polyphenols that were previously shown to impact iron bioavailability [5,7] were detected in cooked beans, which included flavonols, phenolic acids, catechins, and procyanidins (precursors to condensed tannins). High concentrations of kaempferol 3-glucoside were measured in each of the yellow beans ranging from 356 nmol/g in Ervilha to 749 nmol/g in Uyole 98. In contrast to the yellow beans, the kidney beans had little to no kaempferol 3-glucoside after cooking (Table 4). The polyphenol profiles of the two fastest cooking yellow beans Ervilha and Uyole 98 were limited to kaempferol, kaempferol 3-glucoside and kaempferol 3-sumbuioside, while the slower cooking yellow bean PI527538 had significantly (*p* < 0.05) higher concentrations of quercetin 3-glucoside, catechins, and procyanidin B1–B2 (Table 4). Red Hawk had the most diverse set of polyphenols after cooking, including higher concentrations of quercetin 3-glucoside, protocatechuic acid, catechin, and procyanidin B1 (Table 4).

The polyphenol concentrations measured in each of the bean based diets are shown in Table 5. The polyphenol profile of each diet reflects the profiles of the cooked beans, with kaempferol 3-glucoside still being the most dominate polyphenol measured in each of the yellow bean diets. When compared to Ervilha and Uyole 98, significantly (*p* < 0.05) higher concentrations of quercetin 3-glucoside, catechins and procyanidins were detected in PI527538 and Red Hawk bean based diets (Table 5).

### 3.5. In Vitro Iron Bioavailability (Caco-2 Cell Ferritin Formation)

The results in Table 6 show significant (*p* ≤ 0.05) differences in iron bioavailability between the cooked beans and their corresponding diets using the Caco-2 cell bioassay. The bioassay measures ferritin protein formation in cells following exposure to a digested sample that was prepared from either cooked beans or bean based diets on an equal weight basis. Among the cooked beans, significantly (*p* ≤ 0.05) higher iron bioavailability was measured in Ervilha and Snowdon when compared to the Uyole 98, PI527538, and Red Hawk (Table 6). Significantly (*p* ≤ 0.05) higher iron bioavailability was also measured in the Ervilha bean based diet when compared to the other four diets (Table 6).

### 3.6. In Vivo Iron Bioavailability (Gallus gallus Feeding Trial)

#### 3.6.1. Feed and Iron Intakes

**Figure 2:**
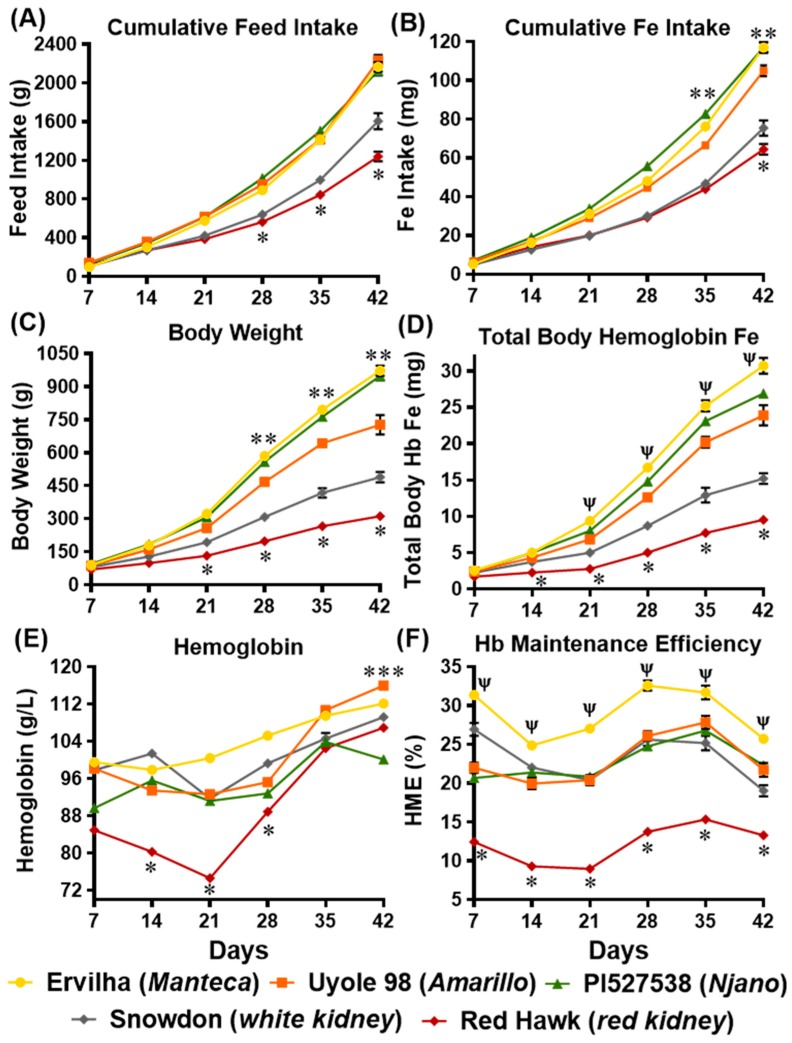
Cumulative feed intake (**A**), Fe intake (**B**), body weight (**C**), total body hemoglobin Fe (**D**), hemoglobin concentration (**E**) and hemoglobin maintenance efficiency (HME); (**F**) during the 6 weeks of consuming bean based diets. Values are means ± SEM (n = 10–13 animals per treatment group). * Significantly (*p* ≤ 0.05) lower values measured in the group receiving the Red Hawk diet. ** Significantly (*p* ≤ 0.05) higher cumulative Fe intakes and higher body weights measured in the groups receiving the Ervilha and PI527538 diets. *** Significantly (*p* ≤ 0.05) higher hemoglobin measured at day 42 in the group receiving the Uyole 98 diet versus the group receiving the PI527538 diet. Ψ Significantly (*p* ≤ 0.05) higher total body hemoglobin Fe and HME values measured in the group receiving the Ervilha diet versus the other four treatment groups.

By day 14 of the study, significantly (*p* ≤ 0.05) higher feed intakes were measured in the groups receiving the yellow bean diets versus the groups receiving the white and dark red kidney diets (Figure 2A). By day 21, the group receiving the Red Hawk diet had significantly (*p* ≤ 0.05) lower feed intakes compared to the other four treatment groups (Figure 2A). Iron intake mirrored the cumulative feed intakes over the course of the experiment. Significant (*p* ≤ 0.05) differences in iron intakes between the five treatment groups could be detected as early as day 7 (Figure 2B). By the end of the study, cumulative iron intakes ranged from 117 mg in the groups receiving the Ervilha and PI527538 yellow bean diets to 64 mg in the group receiving the Red Hawk diet (Figure 2B). Although cumulative feed intakes were not different between the groups receiving the yellow bean diets (Figure 2A), lower iron concentrations in the Uyole 98 diet (47 μg/g) resulted in a significantly (*p* ≤ 0.05) lower iron intake by day 35 of the study-when compared to the groups receiving the Ervilha and PI527538 diets, which had higher iron concentrations (54–55 μg/g; Figure 2B). The specified values for cumulative feed and iron intake are listed with mean separations in Tables S3 and S4.

#### 3.6.2. Growth Rates and Total Body Hemoglobin Iron

Increases in body weights were consistently higher (*p* ≤ 0.05) among the three groups receiving the yellow bean diets when compared the groups receiving the white and red kidney bean diets (Figure 2C). By the end of the experiment, the group receiving the Uyole 98 diet had significantly (*p* ≤ 0.05) lower body weight versus the two groups receiving the Ervilha and PI527538 diets (Figure 2C). Throughout the experiment, the lowest body weights were measured in the group receiving the Red Hawk diet (Figure 2C). Total body hemoglobin iron (Hb-Fe) varied significantly (*p* ≤ 0.05) between the treatment groups throughout the 6 week feeding trial (Figure 2D). By day 21 of the experiment, the group receiving the Ervilha diet had significantly (*p* ≤ 0.05) higher Hb-Fe values when compared to the other four dietary treatments (Figure 2D). Starting at day 7 of the experiment, the lowest Hb-Fe values were measured in the group receiving the Red Hawk diet (Figure 2D). Specified values for body weight and total body hemoglobin iron are listed with mean separations in Tables S5 and S6.

#### 3.6.3. Hemoglobin and Hemoglobin Maintenance Efficiency (HME)

By day 7 of the experiment, the concentrations of hemoglobin (Hb) varied significantly (*p* ≤ 0.05) between the five groups receiving the bean based diets (Figure 2E). From days 14–28 of the experiment, a significant (*p* ≤ 0.05) drop in Hb was measured in the group receiving the Red Hawk diet. The concentrations of Hb in the Red Hawk group recovered by day 35 of the experiment, with no significant differences detected between the other four dietary treatments (Figure 2E). At the end of the experiment, the group receiving the Uyole 98 diet had significantly (*p* ≤ 0.05) higher Hb concentrations than the group receiving the PI527538 diet (Figure 2E). Significant (*p* ≤ 0.05) differences in HME were measured between the five treatment groups starting at day 7 of the experiment (Figure 2F). The group receiving the Ervilha diet had the highest HME percentages throughout the experiment ranging from 25%–33%. The lowest HME percentages (9%–15%) were detected in the group receiving the Red Hawk diet (Figure 2F). Specified values for hemoglobin and hemoglobin maintenance efficiency are listed with mean separations in Tables S7 and S8.

#### 3.6.4. Liver Iron and Ferritin Concentrations

The concentrations of liver iron and ferritin measured at the end of the feeding trial are shown in Table 7. Significant (*p* ≤ 0.05) differences in liver iron were detected among the five treatment groups with concentrations ranging from 64 μg/g in the group receiving the Ervilha diet to 49–51 μg/g in the groups receiving the PI527538 and Red Hawk diets. Significant (*p* ≤ 0.05) differences in liver ferritin concentrations were also measured between the five dietary treatment groups (Table 7). Liver ferritin mirrored liver iron concentrations, ranging from a high of 341 μg/g in the group receiving the Ervilha diet to a low of 110 μg/g in the group receiving the Red Hawk diet. The liver iron and ferritin concentrations between the different treatment groups also mirror the ferritin formation results of the Caco-2 bioassay (Table 6).

#### 3.6.5. Gene Expression of Iron Import and Export Proteins in the Duodenum

**Figure 3:**
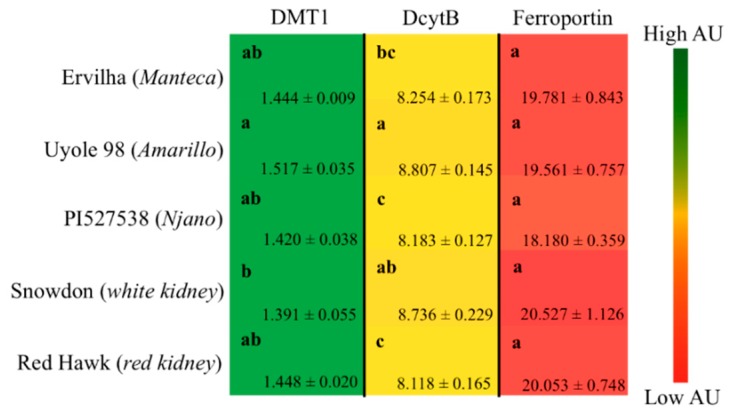
Gene expression of iron proteins in the duodenum after 6 weeks of consuming bean based diets. Values are means ± SEM (*n* = 5 per treatment group). Means sharing the same letter in each column are not significantly different at *p* ≤ 0.05. DMT-1, Divalent Metal Transporter-1; DcytB, Duodenal cytochrome b.

Duodenum gene expression of DMT-1, DcytB and ferroportin relative to 18S rRNA is shown in Figure 3. Significant (*p* ≤ 0.05) differences in the expression of DMT-1 and DcytB were detected, but no significant differences in ferroportin were observed between the five treatment groups (Figure 3). The highest expression levels of DMT-1 and DcytB were detected in the group receiving the Uyole 98 diet (Figure 3). 

#### 3.6.6. Bacterial Populations in the Cecum

**Figure 4:**
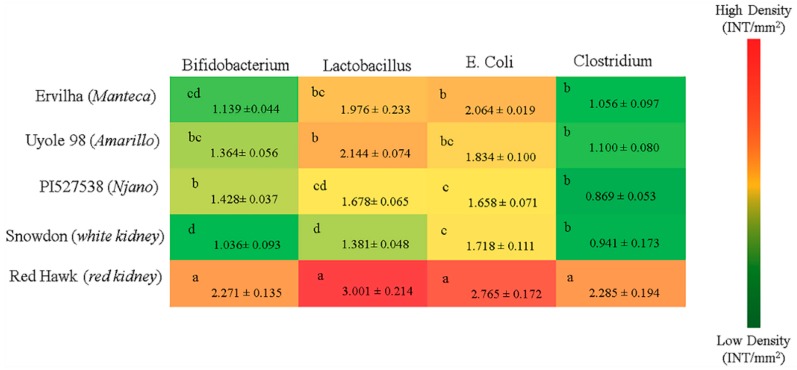
Genera and species-level bacterial populations (AU) from cecal contents after 6 weeks of consuming bean based diets. Values are means ± SEM (n = 6 per treatment group). Means sharing the same letter in each column are not significantly different at *p* ≤ 0.05.

Significant (*p* ≤ 0.05) differences in the relative abundance of all four bacterial populations (Bifidobacterium, Lactobacillus, E. coli, Clostridium) were measured between each of the groups receiving the bean based diets (Figure 4). Low levels of abundance for Bifidobacterium and Lactobacillus were detected in the group receiving the Snowdon diet, when compared to the other treatment groups (Figure 4). Significantly (*p* ≤ 0.05) higher levels of abundance for all four bacterial populations were measured in the group receiving the Red Hawk diet (Figure 4).

## 4. Discussion

### 4.1. Assessing the Iron Bioavailability of African Yellow Beans

Three yellow beans were selected from market classes that would be recognized by consumers in Sub-Saharan Africa [23,24,25]. Two non-yellow bean varieties were also included in this study, each representing a white and red kidney bean produced for commercial food manufacturing. Red beans, such as the dark red kidney, are preferred seed types in many regions of Sub-Saharan Africa and are often marketed alongside yellow beans [23,24]. The white kidney, Snowdon, was included in the study to represent a bean variety with no seed coat polyphenols. Snowdon and Red Hawk are from the same Andean gene pool as the three Africa yellow beans. Snowdon and Red Hawk also share many of the same genetic and agronomic characteristics [30,31]. Previous in vitro and in vivo studies have demonstrated that the iron absorption properties between white and red beans are very different from one another, which creates an ideal frame of reference for comparing the iron bioavailability properties of yellow beans [25,42]. Relative to the each of the yellow beans in this study, both the white and red kidney bean varieties have longer cooking times in boiling water. The overall nutritional value, as well as the iron bioavailability of dry beans is greatly impacted by the conditions in which they are grown, stored, and processed to become edible [25,46,47,48]. To limit these factors, beans were grown under the same field conditions, stored in a controlled environment, and their cooking time was standardized for each genotype to avoid over-or-under processing before diet formulation. 

As previously demonstrated, the Caco-2 bioassay coupled with the *Gallus gallus* in vivo feeding model is an effective two-step system for evaluating the iron bioavailability of staple foods, such as beans [6,38,40,41,42,49]. The Caco-2 bioassay is an initial assessment used to compare varieties and to identify factors in food crops that could potentially impact the absorption of iron in vivo [38,39]. For this study, the bioassay shows that iron uptake (via ferritin formation) in Caco-2 cells was negatively impacted when diets were formulated with the darker colored yellow and red beans (Table 6). The results of the Caco-2 bioassay also matched the same patterns of liver iron and liver ferritin concentrations in each group of animals after the feeding trial (Table 7). Additionally, the bioassay revealed that diets prepared from the Manteca yellow bean produced a higher iron uptake in Caco-2 cells when compared to the other yellow, white and red bean based diets. The bioassay, however, did not predict the precise patterns of iron absorption between Uyole 98, PI527538 and the kidney beans over the course of the in vivo feeding trial. This exemplifies the need for the *Gallus gallus* model to be coupled with the Caco-2 bioassay because total body hemoglobin iron (Hb-Fe) and hemoglobin maintenance efficiency (HME) are two physiological markers that take into consideration an animal’s growth rate, food consumption and adaptation to the bean based diet during the course of a feeding trial [6,40,42,49]. 

The *Gallus gallus* model can also be used to gain insight into the mechanism(s) of iron bioavailability by measuring the hematological, molecular (gene expression) and microbial changes in response to carefully formulated test diets [14,38,40,41,42,50]. Similar to humans, the gene expression of iron absorption proteins and the microbiota profiles of *Gallus gallus* are impacted by dietary iron intake [50,51]. The *Gallus gallus* shares >85% homology with the human gene sequences of the dietary iron import/export proteins DMT-1, DcytB, and ferroportin [52]. In addition, the phylum levels of gut microbiota between *Gallus gallus* and humans are similar, each being dominated by Bacteroidetes, Firmicutes, Proteobacteria and Actinobacteria [14,53,54]. The *Gallus gallus* is the first in vivo model to assess the iron nutrition of yellow beans, and this current study represents the first approach to predict the iron bioavailability of yellow beans in humans [38]. 

The *Gallus gallus* is a useful animal model for testing cooked beans that are combined into diets as either the main ingredient or formulated into a complex meal plan [40,41,42]. For this study, cooked yellow and kidney beans were the main ingredient of bean based diets, contributing to >60% of the total dietary iron in each diet. Bean based diets were formulated with the complementary foods of potato, rice, and cabbage in combinations similar to previous in vivo experiments that have successfully compared the iron bioavailability of different bean varieties in vivo [14,41]. Although the combination of diet ingredients used in this study would be quite common in Sub-Saharan Africa, future studies can be designed to evaluate the iron bioavailability of yellows beans within a multiple ingredient meal plan (the food basket approach), which is more precisely tailored to specific regions where yellow beans are already familiar to consumers [14,41,55].

### 4.2. The Iron Bioavailability of African Yellow Beans 

Of the three African yellow beans tested in this study, the fastest cooking Manteca yellow bean (Ervilha) delivered the most absorbable iron for growth and hemoglobin production during the six week feeding trial. The Manteca is a pale-yellow bean native to Chile, characterized by its gray-black hilum ring. Higher prices at the market and anecdotal claims of ‘low-flatulence’ inspired scientists to examine the Manteca seed type’s biochemistry [20,56,57]. They found the Manteca has a different fiber profile with more digestible starch and protein when compared to other black and red beans [57,58,59]. More importantly, they discovered the Manteca carries a recessive allele that closes off the production of procyanidin (condensed tannin) synthesis in the polyphenol pathway [20,56,60]. 

In addition to producing a darker seed coat color, condensed tannin concentrations influence the cooking time, as well as the digestibility of dry beans [59,61,62]. Although diets formulated with the Ervilha and PI527538 had similar iron concentrations and phytate-iron molar ratios (Table 2), the results of this experiment indicate that the slowest cooking Njano yellow bean PI527538 did not deliver as much iron as the fastest cooking Manteca yellow bean Ervilha. These results are in agreement with the current and past Caco-2 cell culture experiments, which show how Ervilha and PI528537 have distinct iron bioavailability that is independent of their iron concentrations after cooking [25]. The same polyphenols in black and red beans that were previously shown to inhibit the absorption of iron in vitro (quercetin 3-glucoside, procyanidin B1, procyanidin B2) were also detected in PI527538 after cooking [5,6,7,8]. These results provide evidence that the fast cooking Manteca is biochemically unique to other yellow beans, and demonstrates how altering the downstream production of polyphenols to prevent the synthesis of condensed tannins could be a useful mechanism to improve the iron bioavailability of dry beans [5,6,7,8,25]. 

Unlike previous investigations that have only compared the iron bioavailability of non-biofortified and biofortified beans in the same market class [6,14,40,41,55], the results of this study demonstrate that the iron bioavailability of yellow beans after cooking is not dependent on seed iron concentrations alone. For example, total dietary iron concentrations (Table 2) and cumulative iron intakes (Figure 2B) were lower for animals receiving the Amarillo yellow bean Uyole 98 diet, yet they were able to maintain their hemoglobin, Hb-Fe, and HME values, as well as their liver iron concentrations just as effectively as the animals receiving the slower cooking PI527538 yellow bean diet. The up-regulation of the iron import genes DMT-1 and DcytB in the animals receiving the Uyole 98 diet indicates there was a physiological adaptation to the lower dietary iron concentrations [6,40,41]. Despite the changes in the gene expression of iron import proteins, animals receiving the Uyole 98 diet were still unable to maintain their growth and hemoglobin production as efficiently as the animals receiving the fastest cooking Ervilha bean based diet. Interestingly, Uyole 98 had the highest concentrations of kaempferol 3-glucoside, but small amounts of the precursors to condensed tannins (catechin, epicatechin, procyanidin B1) were also detected in Uyole 98 bean based diets. There is evidence to suggest that these compounds may be more potent inhibitors of dietary iron than kaempferol 3-glucoside is a promotor of iron in vitro [5,6,7].

The contrast between Ervilha, Uyole 98, and PI527538 serves as a good example that not all yellow beans will have the same iron bioavailability. Demonstrating that there is phenotypic diversity in iron bioavailability between the different market classes of yellow beans, however, is important for dry bean breeders to identify targets traits that can lead to genetic improvement, while, at the same time, complementing the biofortification efforts that are currently underway to increase raw bean seed iron concentrations in yellow beans [4,63]. This study is the first to show that differences in polyphenols and dietary fiber concentrations between Ervilha, Uyole 98 and PI527538 can also play a role in determining the microbiota populations of the lower intestine (Figure 4). For example, the population densities of Bifidobacterium and E. Coli in the cecum were significantly different between animals fed the Ervilha and PI527538 yellow bean diets. This is an important finding because the polyphenols and fiber (prebiotics) found in dry beans are implicated in maintaining the health of the digestive system by influencing bacterial (probiotic) populations in the lower intestine [9,10,11,12,13,14]. While the measurements of microbiota at the species level is too broad of an interpretation to make any specific health recommendations about yellow beans, a more detailed study is currently being conducted to examine the microbial diversity and compositional changes of gut microbiota at the Phylum and Genus levels in animals fed the yellow and kidney bean based diets.

### 4.3. Iron Bioavailability of the White and Red Kidney Beans

Animals receiving the white and red kidney bean diets had low feed intakes and slower growth rates, which prevented these animals from accumulating the Hb-Fe values that were achieved by the animals receiving yellow bean diets (Figure 2). With the exception of Uyole 98, the white and red kidney beans tested in this study had significantly more dietary fiber and less protein than the African yellow beans (Table 3). There is evidence to support that increases in dietary fiber from habitual pulse consumption increase perceived satiety and reduce food intake, while intervention studies show that pulse consumption leads to reductions in body weight with or without energy restriction [64,65]. The differences in soluble and insoluble fiber concentrations between the two seed types are also indicative of differences in seed coat thickness and cotyledon cell wall composition, which are additional factors that need to be considered when comparing the digestibility and iron bioavailability of yellow and kidney beans [66].

The large differences in Hb-Fe between the animals receiving the white kidney Snowdon diet and the yellow Uyole 98 diet, which had the same iron concentrations, phytate-iron molar ratios, and fiber concentrations, suggest that kaempferol 3-glucoside may facilitate the absorption of dietary iron [5,6,7,8]. The overall digestibility and iron nutrition of African yellow beans with kaempferol 3-glucoside as their most abundant polyphenol appears to be far superior to that of white and red kidney beans-with polyphenol profiles that contain little to no kaempferol compounds. The large differences in growth rates and feed intakes between the two groups receiving the yellow and kidney bean diets, however, makes it difficult to pin-point kaempferol 3-glucoside as the only contributor of improved iron bioavailability among the three African yellow beans. Future investigations are needed to understand kaempferol 3-glucoside’s full potential as a dietary promoter of iron bioavailability in dry beans, as well as in other world food crops that also contain kaempferol compounds, such as the yellow potato or yellow cassava [15].

Comparing the two kidney beans to each other revealed the iron bioavailability of Red Hawk, with a diverse array of polyphenols detected in its dark red seed coat, was significantly lower than Snowdon, which had little to no polyphenols in its white seed coat (Table 4). Animals receiving the Red Hawk diet could not absorb enough iron from the diet to maintain hemoglobin production during the first 2–3 weeks of the feeding trial (Figure 2E). An interesting adaptation in animals receiving the Red Hawk diet, however, was evident between weeks 3 and 4 of the experiment as hemoglobin production began to improve. Of the five beans tested in this study, the highest concentrations of insoluble and soluble fiber were measured in Red Hawk, which is an important observation because a more diverse collection of polyphenols, and more dietary fiber (prebiotics) are important factors that promote the diversity of microbiota populations in the lower intestine [9,10,11,12,13,14]. All four bacterial populations in the cecum at the end of the feeding trial were significantly elevated in the animals receiving the Red Hawk diet, when compared to the animals receiving the white and yellow bean diets (Figure 4). The probiotic microbial activity and fermentation of fiber in the intestinal lumen produce small chain fatty acids (SCFA) that include acetate, propionate, and butyrate [67,68,69]. These molecules are important to the metabolism and health of the digestive system because they promote intestinal cell proliferation and serve as an energy source for colonic epithelial cells [67,68,69]. The recovery of hemoglobin production in the animals receiving the Red Hawk diet by the end of the feeding trial, in conjunction with increased levels of bacteria populations in the cecum, indicates that the microbial composition of the lower intestine can be a contributing factor in the iron status of the host. These results are consistent with previous animal studies that show how dietary fiber can remodel the microbiota in the lower intestine; which in turn, can improve the morphometric parameters of the upper intestine by stimulating the proliferation of enterocytes and the height of duodenal villi [12,13,14,67,69]. These findings continue to support the idea that breeding for specific traits that promote the health and diversity of bacterial populations in the digestive system is an additional strategy that biofortification programs can use to improve the efficacy of biofortified food crops [14,70,71].

## 5. Conclusions

The comparisons between the different market classes of yellow and kidney beans tested in this study provide evidence that the iron bioavailability of yellow beans is unique from other bean color classes, and the important quality traits that distinguish yellows from other seed types at the market place, such as seed coat color and cooking time, are key factors in determining the iron benefits of yellow beans. The yellow bean’s unique bio-delivery of iron provides a new phenotype breeding programs can utilize to improve the iron quality of new biofortified bean varieties, beyond just increasing raw seed iron concentrations [72]. Introducing traits similar to those found in the Manteca yellow bean that reduces the content of procyanidins (condensed tannins), while maintaining the yellow bean’s abundance of kaempferol 3-glucoside, may have a profound impact on dry bean iron bioavailability.

## Figures and Tables

**Table 1:** Description, Sources, Cultivation Status, and Cooking Times of the Five Genotypes Used to Evaluate the Iron Bioavailability of Yellow Beans from Africa and Kidney Beans from North America. ^1^

Name	Seed Type (*Market Class*)	Source	Cultivation	Cooking Time (Min) ^2^
Ervilha	Yellow (*Manteca*)	IIA; Huambo, Angola	Landrace	15.3 ± 0.22 ^e^
Uyole 98	Yellow (*Amarillo*)	Tanzania Breeding	Variety	22.3 ± 0.37 ^d^
PI527538	Yellow (*Njano*)	Burundi; US GRIN	Landrace	26.0 ± 0.63 ^c^
Snowdon	White Kidney	Michigan State Unv.	Variety	29.4 ± 0.37 ^b^
Red Hawk	Dark Red Kidney	Michigan State Unv.	Variety	36.8 ± 0.92 ^a^

^1^ This panel consists of medium to large Andean beans ranging from 58 to 81 g/100 seed. IIA, Instituto de Investigação Agronómica; US GRIN, U.S. Germplasm Resources Information Network. ^2^ Raw seed were soaked in distilled water for 12 h prior to determining the number of minutes to reach 80% cooking time with an automated Mattson pin-drop device. Values are means ± SEM of four field replicates, each measured in duplicate (*n* = 8). Means sharing the same letter are not significantly different at *p* ≤ 0.05.

**Table 2:** Ingredient Formulation, Iron Concentrations and Phytate Analysis of Bean Based Diets.

Ingredient ^1^	Iron	Diet Formulation (g/kg)				
	(μg/g) ^2^	Ervilha	Uyole 98	PI527538	Snowdon	Red Hawk
Ervilha (*Manteca*)	83.0 ± 0.78 ^a^	420	−	−	−	−
Uyole 98 (*Amarillo*)	79.1 ± 0.75 ^b^	−	420	−	−	−
PI527538 (*Njano*)	84.8 ± 0.70 ^a^	−	−	420	−	−
Snowdon (*white kidney*)	75.3 ± 0.50 ^b^	−	−	−	420	−
Red Hawk (*red kidney*)	81.9 ± 0.76 ^a^	−	−	−	−	420
Potato (*white*)	14.6 ± 0.27 ^d^	330	330	330	330	330
Rice (*white/polished*)	6.55 ± 0.54 ^e^	90	90	90	90	90
Cabbage (*white*)	19.8 ± 0.74 ^c^	90	90	90	90	90
Vitamin/mineral premix ^3^	0.00 ± 0.0 ^f^	70	70	70	70	70
*DL*-Methionine	0.00 ± 0.0 ^f^	2.5	2.5	2.5	2.5	2.5
Choline Chloride	0.00 ± 0.0 ^f^	0.75	0.75	0.75	0.75	0.75
*Total Composition (g)*		*1000*	*1000*	*1000*	*1000*	*1000*
**Dietary Analysis ^4^**						
Iron concentration (μg/g)		53.7 ± 1.5 ^a^	46.5 ± 0.36 ^b^	54.5 ± 0.91 ^a^	47.4 ± 0.37 ^b^	52.4 ± 1.1 ^a^
Phytate concentration (mg/g)		7.55 ± 0.22 ^a^	7.30 ± 0.01 ^a^	6.91 ± 0.12 ^b^	7.19 ± 0.11 ^a,b^	7.16 ± 0.08 ^a,b^
Phytate-iron molar ratio		12.1 ± 0.69 ^b,c^	13.9 ± 0.16 ^a^	11.3 ± 0.49 ^c^	12.9 ± 0.92 ^a,b^	12.6 ± 0.86 ^b,c^

^1^ Food ingredients were cooked, drained, and lyophilized prior to milling into a course powder for chemical analysis. ^2^ Values are means ± SEM of five replicates for each ingredient. Means sharing the same letter are not significantly different at (*p* ≤ 0.05). ^3^ Vitamin and mineral premix: #330,002 Chick vitamin mixture; #230,000 Salt mix (no iron) for chick diet (Dyets Inc., Bethlehem, PA, USA). ^4^ Values are means ± SEM of five replicates for each of the bean-based diets. Means sharing the same letter in each row are not significantly different at *p* ≤ 0.05.

**Table 3:** Protein and Fiber Concentrations (g/100 g) of Cooked Beans Used to Formulate Bean Based Diets. ^1^

Cooked Bean	Total Protein	Insoluble Fiber	Soluble Fiber	Total Fiber
Ervilha (*Manteca*)	25.02 ± 0.02 ^b^	17.42 ± 1.20 ^c^	1.99 ± 0.28 ^b^	19.41 ± 1.48 ^c^
Uyole 98 (*Amarillo*)	22.30 ± 0.10 ^c^	19.95 ± 0.32 ^ab^	2.52 ± 0.36 ^ab^	22.47 ± 0.04 ^ab^
PI527538 (*Njano*)	26.05 ± 0.12 ^a^	18.75 ± 0.44 ^bc^	2.09 ± 0.21 ^b^	20.83 ± 0.22 ^bc^
Snowdon (*white kidney*)	21.07 ± 0.14 ^d^	20.71 ± 0.49 ^a^	2.66 ± 0.01 ^a^	23.37 ± 0.50 ^a^
Red Hawk (*red kidney*)	22.83 ± 0.13 ^c^	21.32 ± 0.64 ^a^	2.92 ± 0.45 ^a^	24.24 ± 0.19 ^a^

^1^ Values are means ± SEM (n = 3 replicates). Means sharing the same letter in each column are not significantly different at *p* ≤ 0.05.

**Table 4:** Polyphenol Concentrations (nmol/g) of Cooked Beans. ^1^

Polyphenol	Ervilha	Uyole 98	PI527538	Snowdon	Red Hawk
	(*Manteca*)	(*Amarillo*)	(*Njano*)	(*white kidney*)	(*red kidney*)
Kaempferol	74.0 ± 2.3 ^a^	42.4 ± 5.8 ^b^	40.7 ± 1.5 ^b^	-	1.9 ± 0.3 ^c^
Kaempferol 3-glucoside	356 ± 25 ^c^	749 ± 48 ^a^	671 ± 19 ^b^	0.9 ± 0.1 ^e^	4.7 ± 0.3 ^d^
Kaempferol 3-sambuioside	86.4 ± 7.8 ^a^	40.8 ± 2.5 ^b^	4.4 ± 0.2 ^c^	-	3.7 ± 0.1 ^d^
Quercetin	2.3 ± 0.1 ^c^	-	3.8 ± 0.2 ^b^	-	6.2 ± 0.4 ^a^
Quercetin 3-glucoside	16.2 ± 0.9 ^c^	4.5 ± 0.5 ^d^	57.9 ± 1.3 ^a^	-	23.4 ± 0.8 ^b^
Quercetin 3-rutinoside	-	-	-	-	3.1 ± 0.2
Protocatechuic acid	4.2 ± 0.5 ^c^	6.9 ± 0.5 ^b^	6.8 ± 0.7 ^b^	-	30.6 ± 1.6 ^a^
Catechin	-	2.7 ± 0.2 ^b^	44.4 ± 2.0 ^a^	-	40.0 ± 1.7 ^a^
Epicatechin	-	0.4 ± 0.1 ^c^	8.5 ± 0.8 ^a^	-	5.3 ± 0.3 ^b^
Procyanidin B1	-	3.9 ± 0.3 ^c^	17.4 ± 0.7 ^a^	-	13.7 ± 0.8 ^b^
Procyanidin B2	-	-	1.4 ± 0.1 ^a^	-	0.8 ± 0.2 ^b^

^1^ Values are means ± SEM (*n* = 8 replicates). Means sharing the same letter in each row are not significantly different at *p* ≤ 0.05.

**Table 5:** Polyphenol Concentrations (nmol/g) Measured in Bean Based Diets.^1^.

Polyphenol	Ervilha	Uyole 98	PI527538	Snowdon	Red Hawk
	(*Manteca*)	(*Amarillo*)	(*Njano*)	(*white kidney*)	(*red kidney*)
Kaempferol	7.4 ± 0.3 ^a^	5.6 ± 0.2 ^b^	5.4 ± 0.2 ^b^	-	-
Kaempferol 3-glucoside	153 ± 5.1 ^c^	327 ± 18 ^a^	234 ± 5.9 ^b^	0.6 ± 0.1 ^e^	1.9 ± 0.1 ^d^
Kaempferol 3-sambuioside	37.4 ± 1.6 ^a^	17.6 ± 0.3 ^b^	1.7 ± 0.1 ^c^	-	1.5 ± 0.1 ^c^
Quercetin	-	-	-	-	-
Quercetin 3-glucoside	6.6 ± 0.3 ^c^	1.8 ± 0.1 ^d^	21.4 ± 0.7 ^a^	-	8.2 ± 0.5 ^b^
Quercetin 3-rutinoside	-	-	-	-	3.0 ± 0.2
Protocatechuic acid	2.4 ± 0.4 ^c^	4.5 ± 0.6 ^b^	4.4 ± 0.4 ^b^	-	16.8 ± 1.3 ^a^
Catechin	-	0.8 ± 0.1 ^b^	10.5 ± 0.4 ^a^	-	10.8 ± 0.8 ^a^
Epicatechin	-	0.1 ± 0.0 ^b^	1.7 ± 0.2 ^a^	-	1.3 ± 0.2 ^a^
Procyanidin B1	-	0.7 ± 0.1 ^b^	2.9 ± 0.3 ^a^	-	2.5 ± 0.2 ^a^
Procyanidin B2	-	-	0.2 ± 0.0 ^a^	-	0.2 ± 0.0 ^a^

^1^ Values are means ± SEM (n = 8 replicates). Means sharing the same letter in each row are not significantly different at *p* ≤ 0.05.

**Table 6:** Iron Bioavailability of Cooked Beans and Bean Based Diets Using an in vitro Digestion/Caco-2 Cell Bioassay. ^1^

	Caco-2 Cell Ferritin Formation
Cooked Bean	(ng Ferritin/mg Protein)
Ervilha (*Manteca*)	9.54 ± 0.59 ^a^
Uyole 98 (*Amarillo*)	5.97 ± 0.52 ^b,c^
PI527538 (*Njano)*	5.01 ± 0.15 ^c^
Snowdon (*white kidney*)	8.20 ± 0.50 ^a^
Red Hawk (*red kidney*)	6.92 ± 0.95 ^b^
**Bean Based Diet**	
Ervilha (*Manteca*)	15.5 ± 1.4 ^A^
Uyole 98 (*Amarillo*)	9.46 ± 0.19 ^C^
PI527538 (*Njano*)	7.98 ± 0.80 ^D^
Snowdon (*white kidney*)	12.2 ± 0.41 ^B^
Red Hawk (*red kidney*)	7.57 ± 0.67 ^D^

^1^ In vitro iron bioavailability expressed as Caco-2 cell ferritin concentrations (ng ferritin/mg total cell protein) after a 24 h exposure to an in vitro digestion of either lyophilized cooked beans or bean based diets. Values are means ± SEM of six replicates for each sample. Means sharing the same letter in each group are not significantly different at *p* ≤ 0.05.

**Table 7:** Liver Iron and Ferritin Protein Concentrations After 6 Weeks of Consuming Bean Based Diets. ^1^

Bean Based Diet	Liver Iron	Liver Ferritin
	(μg/g)	(μg/g)
Ervilha (*Manteca*)	64.3 ± 3.7 ^a^	341 ± 14 ^a^
Uyole 98 (*Amarillo*)	54.8 ± 3.4 ^a,b^	243 ± 34 ^b^
PI527538 (*Njano*)	48.6 ± 3.7 ^b^	163 ± 28 ^c^
Snowdon (*white kidney*)	59.1 ± 1.7 ^a,b^	325 ± 4.7 ^a^
Red Hawk (*red kidney*)	51.1 ± 2.3 ^b^	110 ± 18 ^c^

^1^ Values are means ± SEM (n = 10–13 animals per treatment group). Means sharing the same letter in each column are not significantly different at *p* ≤ 0.05. Total iron and ferritin protein concentrations measured as micrograms per gram of liver tissue (wet weight).

## Supplementary Materials

The following are available online at https://www.mdpi.com/2072-6643/11/8/1768/s1, Table S1, Sequences of Forward and Reverse Primers used for PCR Reactions to Measure Gene Expression of Iron Transporter Proteins in the Proximal Duodenum. Table S2, Phytate Concentrations of Each Dietary Component in Bean Based Diets. Table S3, Cumulative Feed Intake During the 6 Weeks of Consuming Bean Based Diets. Table S4, Cumulative Iron Intake During the 6 Weeks of Consuming Bean Based Diets. Table S5, Body Weights During the 6 Weeks of Consuming Bean Based Diets. Table S6, Total Body Hemoglobin Iron (Hb-Fe) During the 6 Weeks of Consuming Bean Based Diets. Table S7, Hemoglobin (Hb) Concentrations. Table S8, Hemoglobin Maintenance Efficacy (HME) During the 6 Weeks of Consuming Bean Based Diets.

## Abbreviations

*P. vulgaris*	*Phaseolus vulgaris* L.
Hb-Fe	total body hemoglobin iron
Hb	hemoglobin
HME	hemoglobin maintenance efficiency
DMT-1	divalent metal transporter-1
DcytB	duodenal cytochrome B
