# Supplementary material for: An In Vivo (Gallus gallus) Feeding Trial Demonstrating the Enhanced Iron Bioavailability Properties of the Fast Cooking Manteca Yellow Bean (Phaseolus vulgaris L.)
Source: Nutrients. 2019 Aug 1;11(8):1768. doi: 10.3390/nu11081768 (PMC6724231; doi:10.3390/nu11081768)
Supplement: Supplementary file 1 [file nutrients-11-01768-s001.pdf]

Table S1. Sequences of Forward and Reverse Primers used for PCR Reactions to Measure Gene Expression of Iron Transporter Proteins in the Proximal Duodenum.

Gene	Forward Primer (5'-3')	Reverse Primer (5'-3")	Base Pair Length	GI Identifier
DMT-1	TTGATTCAGAGCCTCCCATTAG	GCGAGGAGTAGGCTTGTATTT	101	206597489
Ferroportin	CTCAGCAATCACTGGCATCA	ACTGGGCAACTCCAGAAATAAG	98	61098365
DcytB	CATGTGCATTCTCTTCCAAAGTC	CTCCTTGGTGACCGCATTAT	103	20380692
18S rRNA	GCAAGACGAACTAAAGCGAAAG	TCGGAACTACGACGGTATCT	100	7262899

DMT-1, Divalent Metal Transporter-1; DcytB, Duodenal cytochrome B; 18S rRNA, 18S Ribosomal subunit.

Table S2. Phytate Concentrations of Each Dietary Component in Bean Based Diets.[1]

Ingredient	Phytate (mg/g)[2]	Phytate / Iron molar ratio
Ervilha (*Manteca*)	13.7 ± 0.25[a]	14.0 ± 0.39[ab]
Uyole 98 (*Amarillo*)	13.0 ± 0.20[ab]	13.9 ± 0.08[bc]
PI527538 (*Njano*)	13.4 ± 0.05[a]	13.4 ± 0.16[cd]
Snowdon (*white kidney*)	13.6 ± 0.25[a]	15.3 ± 0.38[a]
Red Hawk (*red kidney*)	12.8 ± 0.20[b]	13.4 ± 0.18[d]
Potato (*white*)	1.31 ± 0.01[c]	7.62 ± 0.11[f]
Rice (*white/polished*)	0.76 ± 0.05[e]	9.95 ± 0.52[e]
Cabbage (*white*)	1.08 ± 0.05[d]	4.63 ± 0.37[g]

[1]Values are means ± SEM of five replicates for each ingredient. Means sharing the same letter in each column are not significantly different at $p < 0.05$. [2]Total phytate concentrations measured as milligrams per gram of cooked, drained, lyophilized and milled ingredient (dry weight).

Table S3. Cumulative Feed Intake During the 6 Weeks of Consuming Bean Based Diets.[1]

	Feed Intake (g)					
**Bean Diet**	**Day 7**	**Day 14**	**Day 21**	**Day 28**	**Day 35**	**Day 42**
Ervilha (*Manteca*)	96.30 ± 5.5[b]	300.6 ± 6.3[b]	575.5 ± 1.6[b]	890.7 ± 3.9[b]	1412 ± 9.1[b]	2167 ± 49[a]
Uyole 98 (*Amarillo*)	141.0 ± 4.8[a]	357.3 ± 7.0[a]	615.7 ± 7.6[a]	950.7 ± 8.4[a]	1416 ± 19[ab]	2231 ± 59[a]
PI527538 (*Njano*)	129.7 ± 4.7[a]	342.8 ± 7.1[a]	614.6 ± 7.1[a]	1014 ± 14[a]	1504 ± 23[a]	2127 ± 49[a]
Snowdon (*white kidney*)	104.4 ± 4.3[b]	268.8 ± 5.9 [c]	420.4 ± 4.2[c]	639.2 ± 9.5[c]	995.6 ± 30[c]	1605 ± 84[b]
Red Hawk (*red kidney*)	112.5 ± 1.0[b]	268.7 ± 1.4[c]	384.8 ± 4.9[d]	561.5 ± 10[d]	843.3 ± 18[d]	1239 ± 51[c]

[1]Values are means ± SEM (n = 10 – 13 animals per treatment group). Means sharing the same letter in each column are not significantly different at $p < 0.05$.

Table S4. Cumulative Iron Intake During the 6 Weeks of Consuming Bean Based Diets.[1]

	Iron Intake (mg)					
**Bean Diet**	**Day 7**	**Day 14**	**Day 21**	**Day 28**	**Day 35**	**Day 42**
Ervilha (*Manteca*)	5.200 ± 0.30[bc]	16.23 ± 0.34[b]	31.07 ± 0.09[b]	48.10 ± 0.21[b]	76.23 ± 0.49[b]	117.0 ± 2.4[a]
Uyole 98 (*Amarillo*)	6.628 ± 0.20[a]	16.79 ± 0.38[b]	28.94 ± 0.36[b]	44.68 ± 0.41[b]	66.56 ± 0.93[c]	104.8 ± 2.8[b]
PI527538 (*Njano*)	7.131 ± 0.26[a]	18.86 ± 0.39[a]	33.80 ± 0.94[a]	55.82 ± 0.80[a]	82.37 ± 1.2[a]	117.0 ± 2.7[a]
Snowdon (*white kidney*)	4.907 ± 0.20[c]	12.63 ± 0.28[c]	19.76 ± 0.20[c]	30.04 ± 0.45[c]	46.79 ± 1.4[d]	75.43 ± 3.9[c]
Red Hawk (*red kidney*)	5.850 ± 0.05[b]	13.98 ± 0.07[c]	20.01 ± 0.26[c]	29.20 ± 0.53[c]	43.85 ± 0.96[d]	64.43 ± 2.7[c]

[1]Values are means ± SEM (n = 10 – 13 animals per treatment group). Means sharing the same letter in each column are not significantly different at $p < 0.05$.

Table S5. Body Weights During the 6 Weeks of Consuming Bean Based Diets.[1]

	Weight (g)					
**Bean Diet**	**Day 7**	**Day 14**	**Day 21**	**Day 28**	**Day 35**	**Day 42**
Ervilha (*Manteca*)	89.89 ± 0.51[ab]	179.1 ± 0.33[a]	319.6 ± 4.9[a]	556.2 ± 12[a]	784.5 ± 17[a]	952.4 ± 23[a]
Uyole 98 (*Amarillo*)	86.43 ± 1.3[ab]	161.1 ± 2.4[a]	257.8 ± 7.1[a]	466.9 ± 13[b]	642.9 ± 18[b]	726.6 ± 44[b]
PI527538 (*Njano*)	95.45 ± 2.4[a]	183.8 ± 4.9[a]	308.4 ± 7.0[a]	561.4 ± 13[a]	782.7 ± 17[a]	949.4 ± 21[a]
Snowdon (*white kidney*)	80.82 ± 1.6[b]	128.3 ± 3.2[b]	193.0 ± 9.0[b]	307.6 ± 14[c]	417.8 ± 21[c]	489.1 ± 24[c]
Red Hawk (*red kidney*)	70.11 ± 1.1[c]	98.35 ± 1.4[c]	132.2 ± 5.4[c]	198.5 ± 6.1[d]	265.6 ± 8.8[d]	312.5 ± 12[d]

[1]Values are means ± SEM (n = 10 – 13 animals per treatment group). Means sharing the same letter in each column are not significantly different at $p < 0.05$. Body weights averaged 43 grams at the start of the experiment.

Table S6. Total Body Hemoglobin Iron (Hb-Fe) During the 6 Weeks of Consuming Bean Based Diets.[1]

	Hb-Fe (mg)					
**Bean Diet**	**Day 7**	**Day 14**	**Day 21**	**Day 28**	**Day 35**	**Day 42**
Ervilha (*Manteca*)	2.546 ± 0.02[a]	4.996 ± 0.05[a]	9.367 ± 0.12[a]	16.64 ± 0.34[a]	25.16 ± 0.77[a]	30.72 ± 1.1[a]
Uyole 98 (*Amarillo*)	2.411 ± 0.02[a]	4.285 ± 0.05[b]	6.841 ± 0.31[c]	12.62 ± 0.27[b]	20.23 ± 0.75[b]	23.92 ± 1.4[b]
PI527538 (*Njano*)	2.436 ± 0.06[a]	4.999 ± 0.12[a]	7.986 ± 0.12[b]	14.80 ± 0.25[b]	23.11 ± 0.11[b]	26.93 ± 0.24[b]
Snowdon (*white kidney*)	2.248 ± 0.04[b]	3.710 ± 0.10[c]	5.004 ± 0.19[d]	8.690 ± 0.38[c]	12.87 ± 0.61[c]	15.17 ± 0.75[c]
Red Hawk (*red kidney*)	1.694 ± 0.04[c]	2.265 ± 0.02[d]	2.754 ± 0.11[e]	4.999 ± 0.17[d]	7.720 ± 0.32[d]	9.516 ± 0.31[d]

[1]Values are means ± SEM (n = 10 – 13 animals per treatment group). Means sharing the same letter in each column are not significantly different at $p < 0.05$. Total body hemoglobin iron averaged 0.97 milligrams at the start of the experiment.

Table S7. Hemoglobin (Hb) Concentrations.[1]

Bean Diet	Hb (g/L)					
	**Day 7**	**Day 14**	**Day 21**	**Day 28**	**Day 35**	**Day 42**
Ervilha (*Manteca*)	99.53 ± 0.34[a]	97.83 ± 0.45[a]	100.4 ± 0.37[a]	105.2 ± 0.10[a]	109.5 ± 0.47[a]	112.1 ± 0.51[ab]
Uyole 98 (*Amarillo*)	98.16 ± 0.35[a]	93.46 ± 0.68[a]	92.65 ± 0.54[a]	95.22 ± 0.29[bc]	110.7 ± 0.58[a]	116.0 ± 0.49[a]
PI527538 (*Njano*)	89.67 ± 0.26[a]	95.59 ± 0.51[a]	91.20 ± 0.45[a]	92.83 ± 0.24[bc]	103.9 ± 0.43[a]	100.1 ± 0.43[b]
Snowdon (*white kidney*)	97.79 ± 0.48[a]	101.4 ± 0.31[a]	91.70 ± 0.52[a]	99.26 ± 0.16[ab]	104.6 ± 1.2[a]	109.2 ± 0.62[ab]
Red Hawk (*red kidney*)	84.90 ± 0.35[a]	80.25 ± 0.41[b]	74.58 ± 0.29[b]	88.85 ± 0.25[c]	102.5 ± 0.51[a]	106.9 ± 0.54[ab]

[1]Values are means ± SEM (n = 10 – 13 animals per treatment group). Means sharing the same letter in each column are not significantly different at $p < 0.05$. Concentrations of hemoglobin averaged 79.74 grams per liter at the start of the experiment.

Table S8. Hemoglobin Maintenance Efficacy (HME) During the 6 Weeks of Consuming Bean Based Diets.[1]

	HME (%)					
**Bean Diet**	**Day 7**	**Day 14**	**Day 21**	**Day 28**	**Day 35**	**Day 42**
Ervilha (*Manteca*)	31.36 ± 0.46[a]	24.88 ± 0.23[a]	27.05 ± 0.45[a]	32.60 ± 0.65[a]	31.69 ± 0.87[a]	25.72 ± 0.50[a]
Uyole 98 (*Amarillo*)	22.02 ± 0.66[b]	19.96 ± 0.73[b]	20.42 ± 0.66[b]	26.09 ± 0.60[b]	27.84 ± 0.78[b]	21.74 ± 0.91[b]
PI527538 (*Njano*)	20.68 ± 0.56[b]	21.38 ± 0.40[b]	20.85 ± 0.29[b]	24.75 ± 0.29[b]	26.80 ± 0.22[b]	22.30 ± 0.43[b]
Snowdon (*white kidney*)	26.98 ± 0.86[ab]	22.04 ± 0.31[b]	20.42 ± 0.91[b]	25.64 ± 0.83[b]	25.18 ± 0.92[b]	19.04 ± 0.73[b]
Red Hawk (*red kidney*)	12.44 ± 0.56[c]	9.304 ± 0.21[c]	8.986 ± 0.55[c]	13.76 ± 0.35[c]	15.34 ± 0.44[c]	13.29 ± 0.10[c]

[1]Values are means ± SEM (n = 10 – 13 animals per treatment group). Means sharing the same letter in each column are not significantly different at $p < 0.05$.
